# Achievements, prospects and challenges in precision care for monogenic insulin-deficient and insulin-resistant diabetes

**DOI:** 10.1007/s00125-022-05720-7

**Published:** 2022-05-27

**Authors:** Amélie Bonnefond, Robert K. Semple

**Affiliations:** 1 Inserm UMR1283, CNRS UMR8199, European Genomic Institute for Diabetes (EGID), Institut Pasteur de Lille, Lille University Hospital, Lille, France; 2 Université de Lille, Lille, France; 3 Department of Metabolism, Imperial College London, London, UK; 4 Centre for Cardiovascular Science, University of Edinburgh, Edinburgh, UK; 5 MRC Human Genetics Unit, Institute of Genetics and Cancer, University of Edinburgh, Edinburgh, UK

**Keywords:** Diabetes, Genetics, Insulin resistance, Leptin, Lipodystrophy, MODY, Neonatal diabetes, Personalised medicine, Precision medicine, Review, Sulfonylurea, Thiazolidinedione

## Abstract

Integration of genomic and other data has begun to stratify type 2 diabetes in prognostically meaningful ways, but this has yet to impact on mainstream diabetes practice. The subgroup of diabetes caused by single gene defects thus provides the best example to date of the vision of ‘precision diabetes’. Monogenic diabetes may be divided into primary pancreatic beta cell failure, and primary insulin resistance. In both groups, clear examples of genotype-selective responses to therapy have been advanced. The benign trajectory of diabetes due to pathogenic *GCK* mutations, and the sulfonylurea-hyperresponsiveness conferred by activating *KCNJ11* or *ABCC8* mutations, or loss-of-function *HNF1A* or *HNF4A* mutations, often decisively guide clinical management. In monogenic insulin-resistant diabetes, subcutaneous leptin therapy is beneficial in some severe lipodystrophy. Increasing evidence also supports use of ‘obesity therapies’ in lipodystrophic people even without obesity. In beta cell diabetes the main challenge is now implementation of the precision diabetes vision at scale. In monogenic insulin-resistant diabetes genotype-specific benefits are proven in far fewer patients to date, although further genotype-targeted therapies are being evaluated. The conceptual paradigm established by the insulin-resistant subgroup with ‘adipose failure’ may have a wider influence on precision therapy for common type 2 diabetes, however. For all forms of monogenic diabetes, population-wide genome sequencing is currently forcing reappraisal of the importance assigned to pathogenic mutations when gene sequencing is uncoupled from prior suspicion of monogenic diabetes.

**Graphical abstract:**

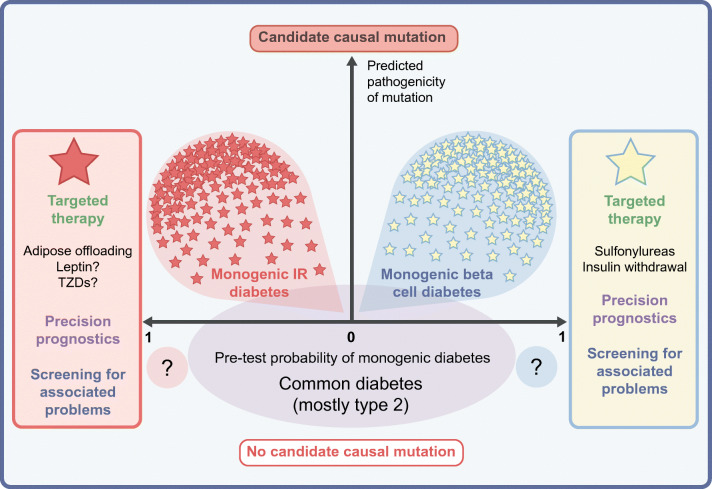

**Supplementary Information:**

The online version of this article (10.1007/s00125-022-05720-7) contains a slideset of the figures for download, which is available to authorised users.

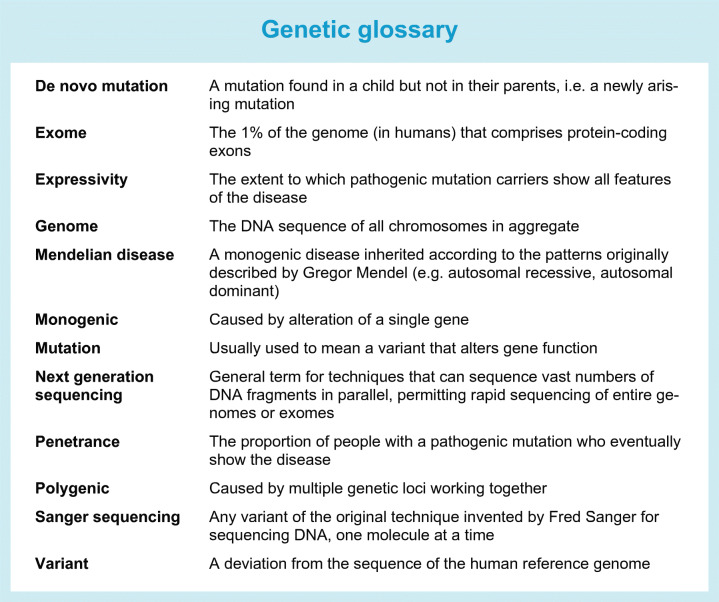



## Background

Diabetes mellitus affects around 9.3% of the world population [1]. In the USA, 91% of diabetes is classified as type 2 diabetes, 6% as type 1 diabetes and 3% as ‘other forms’ [2]. Continuing efforts aim to stratify type 1 and type 2 diabetes into subtypes that inform therapy, the sine qua non of precision diabetes care; however, progress to date has not translated into significant changes in mainstream care. In contrast, study of diabetes caused by single gene mutations has transformed treatment for many patients, in a triumph of the precision medicine approach.

Dominantly inherited, early onset diabetes was first reported in the 1970s [3], a time when unusually severe forms of insulin resistance were also attracting scrutiny [4]. The first identification of a genetic basis for severe insulin resistance—mutations in the gene encoding the insulin receptor (*INSR*)—was in 1988 [5, 6], 3 years after sequencing of the *INSR* gene. *GCK* was the first gene established to cause so-called ‘MODY’, using traditional genetic mapping in 1992 [7, 8]. There has since been a rapid pace of discovery of further monogenic forms of diabetes, reinvigorated by application of next generation sequencing (NGS) over the past 12 years. For several subtypes of monogenic diabetes, distinct therapeutic responses have been demonstrated.

Despite these advances, there is still much work to be done to ensure that all patients with monogenic diabetes receive a timely diagnosis and, where appropriate, targeted therapy. The barriers to achieving this are paradoxically opposed. The high prevalence of diabetes means that diabetes service organisation has focused on efficient, high-throughput systems of care, built around algorithms derived from large clinical trials in type 1 and type 2 diabetes. This is at odds with the attention to detail and bespoke diagnostic testing required for monogenic diabetes, and leads to the problem of clinical underdiagnosis in many settings.

On the other hand, the rapid advance of NGS, including its deployment in population-based studies and its commercial provision directly to consumers, has yielded a surfeit of genetic information. This is often acquired without consideration of pretest probability of a monogenic condition. Attenuation of the traditional pathway from clinical assessment to targeted genetic analysis is sometimes compounded by insufficiently stringent algorithms to discriminate disease-causing mutations from multitudes of irrelevant variants. This risks genetic overdiagnosis in other settings [9–11]. In fact, ascertainment of cases purely through gene sequencing rather than by clinical assessment and targeted testing reduces penetrance and expressivity even for many established pathogenic variants [12, 13].

These developments pose challenges as well as opportunities for genetic stratification of diabetes, and demand regular re-evaluation of strategies for genetic testing in diabetes clinics. We now appraise the current implementation of precision medicine for monogenic diabetes, building on recent comprehensive treatment of monogenic beta cell diabetes in *Diabetologia* [14].

## Monogenic pancreatic beta cell defects

**Fig. 1:**
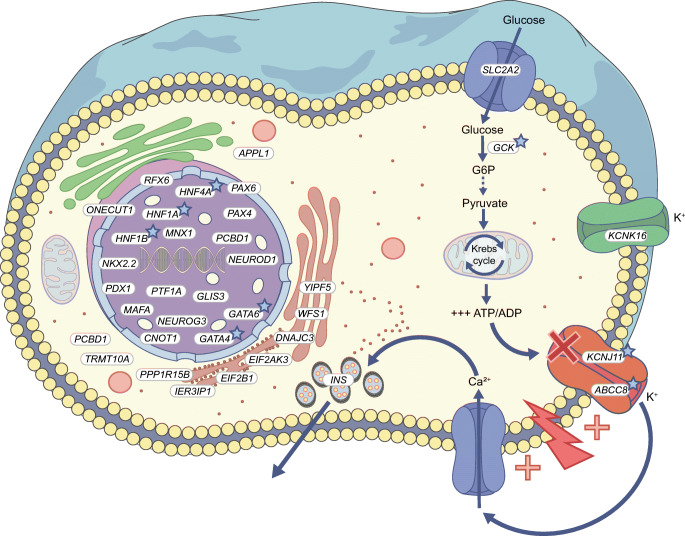
Genes linked with monogenic insulin-deficient diabetes encode proteins that play a key role in pancreatic beta cells. Genes marked with a light-blue star are actionable monogenic beta cell diabetes genes. The dashed line between G6P and pyruvate indicates that several steps are involved. G6P, glucose-6-phosphate. This figure is available as part of a downloadable slideset

Failure of pancreatic beta cells to secrete sufficient insulin to return high blood glucose rapidly to baseline is necessary and sufficient for diabetes to occur. Nearly all genes implicated in monogenic, non-autoimmune, insulin-deficient diabetes correspondingly encode proteins with roles in pancreatic beta cell development and/or function (Fig. 1). Monogenic, insulin-deficient diabetes encompasses rare neonatal diabetes (diagnosed under 6 months of age), more frequent MODY (usually presenting before 25 years of age) and rare recessive syndromes mostly observed in consanguineous families. Pathogenic mutations in more than 30 genes have been found to cause monogenic beta cell diabetes to date, initially using linkage or homozygosity analyses in families or Sanger sequencing of candidate genes, with NGS (whole-exome or whole-genome) [15] more recently dominating.

Genes implicated in monogenic diabetes include several encoding transcription factors involved in pancreatic beta cell development (e.g. *HNF1A*, *HNF1B*, *HNF4A*, *PDX1*, *GATA4*, *GATA6*). Interestingly, diabetes is not simply a consequence of loss of beta cell differentiation in all cases, however. In people with MODY due to *HNF4A* mutations, early life hypoglycaemia due to insulin hypersecretion is also described, and may be sustained [16], while 7% of those deficient for *HNF1A* develop liver adenomatosis [17]. These observations suggest dysregulation rather than loss of endodermal development to beta cells.

Several more beta cell diabetes genes encode endoplasmic reticulum proteins that enable large-scale synthesis of insulin by beta cells (e.g. *WFS1*, *DNAJC3*, *EIF2B1*, *EIF2AK3*), and the gene encoding insulin itself (*INS*). Crucially for precision therapy, two further genes encode the core and regulatory subunits of a key ATP-sensitive potassium channel (*KCNJ11* and *ABCC8*, respectively), while one encodes glucokinase (*GCK*). These three genes play vital roles in the sensing of blood glucose and the transducing of glucose concentration into insulin secretion (Fig. 1). Heterozygous mutations in *GCK* or *HNF1A* are the commonest causes of monogenic diabetes in European populations.

Autosomal recessive syndromes featuring insulin deficiency include Wolfram syndrome (*WFS1* mutations), Mitchell–Riley Syndrome (*RFX6*) and Wolcott–Rallison syndrome (*EIF2AK3*) (Table 1). NGS in suspected MODY without syndromic features has shown some such people to harbour mutations in other genes implicated in syndromic diabetes [18–21]. Such blurring of the demarcation between syndromic and non-syndromic diabetes and between different Mendelian inheritance patterns is emerging across monogenic beta cell and insulin-resistant diabetes.

**Table 1:** Examples of complex diabetes syndromes

Syndrome	OMIM identifier	Gene	Inheritance	Selected syndromic features	Cell structure or process affected
Insulin-deficient diabetes					
Wolfram syndrome	222300	*WFS1*	AR	Optic atrophy Deafness Diabetes insipidus	Unfolded protein response
Wolcott–Rallison syndrome	226980	*EIF2AK3*	AR	Facial dysmorphism Skeletal dysplasia Short stature	Unfolded protein response
Mitchell–Riley syndrome	615710	*RFX6*	AR	Pancreatic hypoplasia Intestinal atresia Gallbladder hypoplasia	Selected gene transcription
Mitochondrial diabetes	520000	Various mitochondrial DNA mutations	Maternal	Deafness Variable multisystem features	Mitochondria
Insulin-resistant diabetes					
SHORT syndrome	269880	*PIK3R1*	AD	Short stature Ocular abnormalities Facial dysmorphism	Insulin/IGF signalling
Mandibuloacral dysplasia	248370 608612	*LMNA* *ZMPSTE24*	AR	Short stature Mandibular hypoplasia Acro-osteolysis of clavicle, phalanges Skin atrophy	Nuclear lamina
Hutchinson–Gilford and atypical progeria syndromes	176670	*LMNA*	AD	Short stature Mandibular hypoplasia Alopecia Osteoporosis Premature ageing Early vascular disease	Nuclear lamina
Werner syndrome	277700	*WRN*	AR	Premature ageing Early cancer	DNA replication and repair
Bloom syndrome	210900	*BLM*	AR	Short stature UV hypersensitivity	DNA replication and repair
MDPL syndrome	615381	*POLD1*	De novo/AD	Mandibular hypoplasia Deafness Progeroid features Lipodystrophy	DNA replication and repair
Seckel syndrome 10	617253	*NSMCE2*	AR	Dwarfism Facial dysmorphism Ovarian failure	DNA replication and repair
Alström syndrome	203800	*ALMS1*	AR	Retinal degeneration Deafness Cardiomyopathy	Centrosome
Osteodysplastic primordial dwarfism of Majewski type 2	210720	*PCNT*	AR	Dwarfism Facial dysmorphism Skeletal dysplasia Shallow teeth	Centrosome
SOFT syndrome	614813	*POC1A*	AR	Short stature Onychodysplasia Facial dysmorphism Hypotrichosis	Centrosome

AR, autosomal recessive; AD, autosomal dominant; MDPL, mandibular hypoplasia, deafness, progeroid features, lipodystrophy; OMIM, Online Mendelian Inheritance in Man; SHORT, short stature, hernia, ocular depression, Rieger’s anomaly, teething delay; SOFT, short stature, onychodysplasia, facial dysmorphism and hypotrichosis; UV, ultraviolet

### Precision therapy in monogenic beta cell diabetes

Identification of monogenic diabetes genes has allowed ground-breaking advances in precision medicine [14], many focused on the cheap sulfonylurea drugs long used in type 2 diabetes. Sulfonylureas bind and inhibit the *ABCC8*-encoded regulatory subunit of the hyperpolarising Kir6.2 potassium channel in beta cells. Mutations in *KCNJ11* or *ABCC8* that increase the opening probability of the channel hyperpolarise the beta cell membrane and inhibit insulin secretion, causing either neonatal diabetes or MODY. Affected patients, remarkably, can often stop insulin therapy on genetic diagnosis and transfer safely onto long-term sulfonylurea treatment [22–26].

People with MODY and heterozygous *HNF1A* or *HNF4A* mutations are also highly sensitive to sulfonylureas. Mouse studies suggested this is accounted for by delayed clearance of sulfonylureas [27], but this appears not to hold in humans [28]. Initial case reports were followed up by larger case series, and it is now established that people deficient for *HNF1A* or *HNF4A* are optimally treated with oral sulfonylureas, possibly with an adjunctive dipeptidyl peptidase 4 inhibitor or glucagon-like peptide 1 receptor agonist [29, 30].

Success in treatment changes after genetic diagnosis is neither certain nor always sustained. Standards for interpretation of variants in rare genetic disorders [31] must first be applied, so that treatment change is only attempted in appropriate genetic contexts. In a recent study, among 16 patients with MODY who were offered treatment switch after genetic diagnosis, nine (six *HNF1A*, three *KCNJ11*) were stably switched from insulin to oral sulfonylurea but seven (three *HNF4A*, two *ABCC8* and one each *HNF1A* and *KCNJ11*) had to restart insulin [32]. The strongest predictor of stable transfer was a high plasma C-peptide concentration, with younger age, lower HbA_1c_ and shorter duration of diabetes of lesser importance [32]. The last two factors, together with low BMI, predicted successful treatment change in another study focused on patients deficient for *HNF1A* or *HNF4A* [33]. These findings emphasise the importance of developing diagnostic pathways to identify patients potentially treatable with sulfonylureas early in disease progression.

Sometimes precision medicine involves avoidance of therapy in selected subgroups. Thus, people with MODY with mild to moderate hyperglycaemia solely due to *GCK* deficiency do not require glucose-lowering treatment. In this case the problem is altered beta cell glucose sensing, raising the homeostatic set point for glucose, and causing lifelong mild, non-progressive hyperglycaemia. People with *GCK*-MODY have been shown not to develop severe complications. Indeed, the prevalence of nephropathy and macrovascular complications in 99 patients with MODY deficient for *GCK* was found to be similar to the prevalence in people without diabetes despite nearly 50 years of hyperglycaemia [34]. While the prevalence of retinopathy was significantly increased in *GCK*-deficient individuals compared with control groups (30% vs 14%), they did not require laser therapy [34]. Hypoglycaemic agents are generally ineffective in *GCK* deficiency, likely due to the power of the homeostatic loop maintaining blood glucose concentration: among 799 patients with *GCK*-MODY, HbA_1c_ was similar in patients on pharmacological treatment (oral agents or insulin) and those on no therapy [35]. Furthermore, among 16 patients, discontinuation of therapy for at least 3 months did not alter HbA_1c_ [35].

### Monogenic insulin-deficient diabetes as the sentinel feature of wider syndromes

A final important aspect of precision diabetes is that for some genetic alterations diabetes may be the sentinel feature of a wider spectrum of abnormalities [14]. This is unsurprising given the important roles in visceral development played by MODY transcription factors, and given fundamental cellular functions of recessive beta cell diabetes genes (Fig. 1). For example, patients with diabetogenic mutations in *HNF1B*, *GATA4*, *GATA6*, *WFS1* or mitochondrial DNA often have developmental anomalies beyond the pancreatic islets, including pancreatic agenesis; kidney, genital tract and heart malformations; and deafness or hearing loss, all showing variable penetrance. Among 201 patients with an *HNF1B* mutation, one study showed that while 82% presented with diabetes, 44% also had stage 3–4 renal impairment and 21% end-stage renal disease at diagnosis [36]. Furthermore, among 102 patients who eventually developed diabetes and renal disease, kidney dysfunction was diagnosed before diabetes in 39%, diabetes and kidney dysfunction were diagnosed concomitantly in 24% and kidney dysfunction was diagnosed after a median diabetes duration of 11 years in 37% [36]. Patients with *GATA6* or *GATA4* mutations can present with a wide spectrum of diabetic presentations and/or cardiac malformation, even within the same family [37–39].

Variable penetrance for components of complex syndromes is common, and widely ascribed to differing genetic backgrounds. Indeed, a high genome-wide burden of common risk alleles from genome-wide association studies (GWAS) confers a risk of diseases including diabetes that approaches the risk of single pathogenic Mendelian mutations [40]. Moreover, the penetrance of diabetes in those with *HNF1A* mutations is significantly influenced by polygenic type 2 diabetes risk score [41, 42]. Interactions among rarer alleles are also likely, but adequately powered studies to identify genetic modifiers agnostically in rare disease are notoriously challenging or impossible for all but the least rare disease-causing mutations.

### Cost-effectiveness of genetic testing in monogenic insulin-deficient diabetes

Genetic screening for monogenic diabetes has been found to be highly cost-effective in economic analyses in the USA based on contemporary incremental cost-effectiveness ratio thresholds [43, 44]. These studies, published before the advent of NGS, modelled: (1) testing for mutations in *KCNJ11* or *ABCC8* in hypothetical 6-year-old patients with permanent neonatal diabetes; or (2) testing for mutations in *HNF1A*, *HNF4A* or *GCK* in hypothetical newly diagnosed MODY patients at 25 to 40 years of age, otherwise presumed to have type 2 diabetes. More recently, simulation of routine screening for MODY based on NGS (targeting *GCK*, *HNF1A*, *HNF4A*, *ABCC8* and *KCNJ11*) in all paediatric diabetes, including children with presumed type 1 diabetes, found that this could significantly reduce health system costs and improve patient quality of life in Australia [45].

## Adipose tissue and insulin signalling defects

Single gene disorders causing severe insulin resistance are the complement of beta cell disorders. Unlike beta cell diabetes, where hyperglycaemia occurs early, it may be delayed or even absent in monogenic severe insulin resistance. This is because pancreatic islets have a large capacity to respond to severe insulin resistance through beta cell hyperplasia and insulin hypersecretion. It is not uncommon for people with insulin receptor (*INSR*) defects to maintain plasma insulin concentrations 1–2 orders of magnitude above normal for years before diabetes develops. Before this, however, morbidity is common, driven by effects of hyperinsulinaemia on ovaries, skin and other soft tissues. In lipodystrophy, ‘lipotoxic’ complications including fatty liver, dyslipidaemia and their sequelae also usually precede diabetes [46]. First presentation of severe insulin resistance may thus be to lipid, endocrinology, liver, gynaecology, dermatology, surgical or other services. All clinicians should be alert to the value of acanthosis nigricans, a velvety browning and thickening of flexural skin, as a sign of insulin resistance. Finding this in lean patients is an important indicator of possible monogenic insulin resistance.

**Fig. 2:**
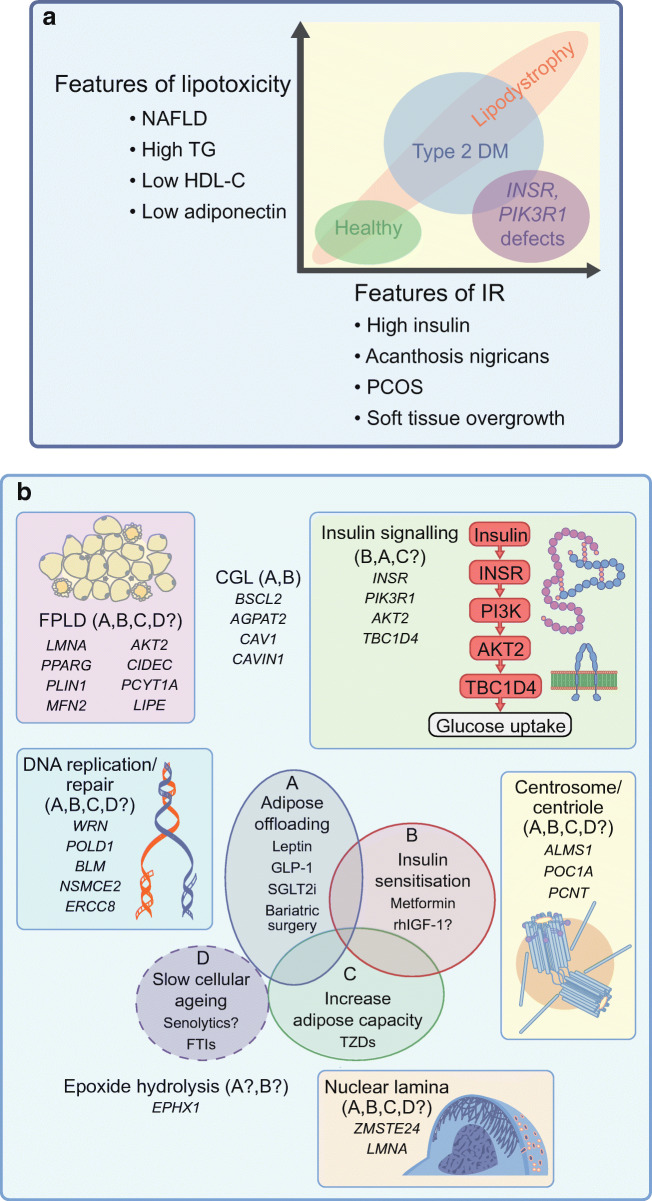
Monogenic insulin resistance subtypes and therapeutic strategies. (**a**) Clustering of severe insulin resistance subtypes according to severity of insulin resistance and lipotoxic features, with reference to health and type 2 diabetes (Type 2 DM). HDL-C, HDL-cholesterol; IR, insulin resistance; NAFLD, non-alcoholic fatty liver disease; PCOS, polycystic ovary syndrome; TG, triacylglycerol. (**b**) Genes implicated in monogenic severe insulin resistance of different subtypes and potential therapeutic strategies. Possible strategies, supported by case series and clinical experience for each subtype, are indicated in brackets, with reference to the Venn diagram (and labelled A–D). Senolytic therapies are a possible future prospect only. CGL, congenital generalised lipodystrophy; FPLD, familial partial lipodystrophy; FTIs, farnesyl transferase inhibitors; SGLT2i, sodium−glucose cotransporter 2 inhibitor. This figure is available as part of a downloadable slideset

Over 33 years since pathogenic mutations in the *INSR* gene were discovered in people with extreme insulin resistance, more than 25 monogenic disorders that feature insulin resistance disproportionate to body fat mass have been described (Table 1, Fig. 2). In this discussion, genetic disorders where insulin resistance is secondary to severe obesity, and appears wholly explained by the degree of obesity, will not be considered, as they have been well reviewed elsewhere [47].

When efforts began to establish the genetic aetiology of severe insulin resistance, the parsimonious assumption was that most causal genes would encode components of the insulin signalling pathway that was then being elucidated. However, such defects have proved surprisingly rare. Apart from *INSR* defects, only a handful of other bona fide signalling defects have been found, including a loss-of-function mutation in *AKT2*, a key intracellular mediator of insulin action, in a single family [48], and dominant mutations affecting another critical signalling enzyme, phosphoinositide 3-kinase (PI3K), in multiple families [49]. Rare heterozygous mutations in *TBC1D4* [50], which regulates insulin-responsive glucose transport, also may cause severe insulin resistance.

Monogenic insulin resistance is now known to be caused much more commonly by defects in development or maintenance of adipose tissue [46], which produce lipodystrophy. In these clinically and genetically heterogeneous disorders, adipose defects may be generalised or partial, and inheritance may be recessive or dominant. Inherited lipodystrophies have recently been comprehensively reviewed elsewhere [51].

As well as relatively ‘clean’ defects in insulin action or adipose tissue development/function, a wide range of rare disorders have been described where severe insulin resistance is part of a more complex syndrome (Table 1, Fig. 1). Some recurring themes have emerged in cellular organelles or pathways affected in such disorders. More than one causal gene is involved in function of the nuclear lamina, an external scaffold for the nucleus (*LMNA* [52], *ZMPSTE24* [53]), in formation of cell membrane invaginations known as caveoli (*CAVIN1* [54], *CAV1* [55]), in centrosomal function (e.g. *ALMS1* [56], *POC1A* [57], *PCNT* [58]) or in DNA replication and/or repair (e.g. *WRN* [59], *POLD1* [60], *NSMCE2* [61]) (Table 1, Fig. 2). These findings imply vulnerability of adipose tissue to certain types of DNA damage or perturbation of cell division, in keeping with features of accelerated ageing seen in several of the syndromes (Table 1). Most of these syndromes are diagnosed early based on characteristic developmental abnormalities; however, for some disorders diabetes may be the sentinel presentation, either due to late onset of other syndromic features, as in Werner syndrome [59], or in formes frustes of classical syndromes. As for syndromic beta cell defects, it is important that diabetologists are vigilant for patterns of abnormalities associated with diabetes that give clues to a genetic cause.

### Precision therapy in monogenic insulin resistance

Identifying monogenic insulin resistance has several implications for therapy, although evidence rests on uncontrolled case series and physiological rationales rather than randomised, controlled trials. In severely insulin-resistant diabetes plasma insulin concentrations are usually extremely high, and therapy with insulin-sensitising agents is strongly favoured over secretagogues such as sulfonylureas. Patients with monogenic insulin resistance also usually require more multidisciplinary care than is common in diabetes clinics. This may address subfertility and hyperandrogenism driven by insulin resistance and common in all monogenic insulin resistance [62], the severe fatty liver disease and dyslipidaemia seen in lipodystrophy, other manifestations of soft tissue overgrowth or syndromic features of complex insulin resistance. Some forms of precision therapy are specific to monogenic insulin resistance subtypes.

#### Insulin signalling defects

In extreme insulin resistance due to recessive *INSR* defects, uncontrolled case series indicate that recombinant human IGF-1 (rhIGF-1), a homologue of insulin, or leptin, discussed below, exerts acute and chronic benefits [63, 64]. Although rhIGF-1 has also occasionally been used in older patients with heterozygous *INSR* defects [63, 65], the long-term risks and benefits of these agents are unclear, and they have no place in therapy outside clinical trials in this group. Reactivation of some mutant *INSR* using monoclonal antibodies has been tested in models [66, 67], and has promise for the future, but remains experimental.

The lack of dyslipidaemia or fatty liver in severe insulin resistance due to *INSR* or *PIK3R1* defects [68, 69] raises the possibility that these subtypes of insulin-resistant diabetes might confer lower macrovascular risk than type 2 diabetes, mandating a different, ‘precision’ approach to primary prevention; however, this has not been tested longitudinally (Fig. 2a).

#### Lipodystrophy

Lipodystrophic insulin resistance currently presents the greatest opportunity for precision therapy in monogenic insulin-resistant diabetes. Sustained positive energy balance, common in contemporary society, normally leads to sequestration of excess energy in adipose tissue as energy-dense triacylglycerol, and ultimately obesity. In lipodystrophy, however, adipose tissue is absent or has impaired ability to store energy. ‘Adipose failure’ thus occurs early when adipose storage is called upon [70, 71], and progression to insulin resistance and diabetes is dramatically accelerated, commonly being seen even in those with normal BMI. This is most severe in those with no adipose tissue (generalised lipodystrophy), who often develop extreme ‘lipotoxic’ insulin resistance, featuring hypertriacylglycerolaemia, pancreatitis, fatty liver disease and early atherosclerosis, despite being lean and often athletic in appearance.

This exquisite sensitivity to energy excess means that reversing positive energy balance in lipodystrophy is crucial. This is achieved by treating affected patients as having ‘obesity’ complications despite normal or low BMI. Adipose tissue offloading measures include hypoenergetic, low-fat diet, and therapies normally limited to obese patients, including glucagon-like peptide-1 (GLP-1) agonists or bariatric surgery [72] (Fig. 2b). The adipose offloading strategy is validated by observation of people with lipodystrophy who do sustain neutral energy balance, for example, through intensive endurance sport. Where no demand is placed on adipose tissue, such individuals can remain entirely metabolically healthy, in a state of latent lipodystrophy.

Lipotoxicity is particularly intractable when adipose tissue is absent or nearly absent, and blood concentrations of the adipose-derived hormone leptin are low or undetectable. This hyperactivates hypothalamic appetite centres, producing intense hunger and increased food intake. This targets the physiological weak point—impaired adipose storage capacity—in a vicious circle. Mitigating this drive to eat is likely to be the main reason for efficacy of recombinant human leptin injection in lipodystrophy when baseline leptin concentration is particularly low (e.g. [73–75]). Leptin has shown major metabolic benefits in uncontrolled trials in generalised lipodystrophy, and lesser benefit in severe partial lipodystrophy. It is licensed in Europe for both subtypes when metabolic control remains poor despite best available treatment, and in the USA for generalised lipodystrophy only. Even the lowest concentrations of leptin may be seen in metabolically healthy lean people, especially prepubertally, and so plasma leptin concentration alone cannot serve as an indication for replacement. Instead, it must be viewed in the context of overall metabolic state. In lipotoxic insulin resistance a plasma leptin threshold for replacement around 4 μg/l was initially suggested [76], but later analysis did not confirm a robust threshold [77]. Workup in an experienced centre is thus highly desirable before leptin therapy is considered.

A complementary strategy to offloading adipose tissue would, in principle, be to increase adipose storage capacity (Fig. 2b). The nuclear hormone receptor peroxisome proliferator-activated receptor-γ (PPARγ), encoded by *PPARG*, is the master regulator of adipose tissue biogenesis, and its pharmacological activation by pioglitazone or other thiazolidinediones (TZDs) seems an obvious strategy in lipodystrophy. Many reports attest to metabolic benefits of TZDs in partial lipodystrophies (e.g. [78, 79]), but they are often disliked by patients as they increase the size of non-affected adipose tissue depots [80], for example, around the head and neck or on the medial thighs. Moreover, the exquisite dietary sensitivity of people with lipodystrophy complicates efforts to discriminate beneficial effects of pharmacotherapy in uncontrolled observational studies, as detection of drug effects may be confounded by effects of concomitant behavioural alterations.

TZDs have attracted specific interest in familial partial lipodystrophy type 3 (FPLD3), the second commonest monogenic lipodystrophy, which is caused by mutations in the *PPARG* gene itself [81, 82]. A priori, it was thought that TZDs might either be particularly effective in FPLD3, by targeting the causal defect, or particularly ineffective, if causal *PPARG* mutations were unresponsive to agonist therapy. This question is not yet answered by trials, but at least some *PPARG* variants respond in cellular studies to potent exogenous TZDs, sometimes when they fail to respond to putative endogenous ligands [83].

Uniquely among genes causing monogenic insulin resistance, *PPARG* has been subject to ‘saturation’ mutagenesis coupled to massively parallel assay of mutation consequences. This has produced experimental evidence for the functional consequences of the large majority of possible *PPARG* missense mutations [84], and has suggested that up to one in 500 people harbour a *PPARG* loss-of-function mutation. This sets the stage for more formal testing at scale of precision TZD therapy in FPLD3, and provides a paradigm with potential applicability to many other forms of monogenic diabetes.

#### Complex insulin resistance syndromes

Several complex syndromes feature insulin-resistant diabetes with frank lipodystrophy, or resemble lipodystrophy metabolically with lesser degrees of abnormal adipose distribution. It is reasonable to assume that the principles of management of lipodystrophy apply to these disorders. No more precision therapies are yet proven to be efficacious for metabolic endpoints, although the US Food and Drug Administration (FDA) recently licensed lonafarnib, a farnesyltransferase inhibitor that reduces accumulation of damaging fragments of lamin proteins, in Hutchinson–Gilford syndrome, a rare premature ageing disorder caused by defects in the *LMNA* gene [85]. As lipodystrophy is a component of many laminopathies, it is plausible that this targeted therapy will also have metabolic benefits in selected patients. Other prospects include senolytic therapies that target senescent cells in adipose tissue [86]. These may be particularly worthy of evaluation in syndromes featuring defective DNA damage sensing and/or repair or impaired centrosomal function (Fig. 2b, Table 1).

### Cost-effectiveness of testing for and treating monogenic insulin-resistant diabetes

Few health economic analyses of either screening for or treatment of monogenic insulin-resistant diabetes have been undertaken. The high cost of recombinant human leptin therapy (the UK National Health Service [NHS] indicative price for adults starts at £200,000 to 400,000 annually) meant that economic modelling was important prior to approval of leptin therapy in lipodystrophy by the UK National Institute for Health and Care Excellence [87]. This modelling included quality of life as well as metabolic control. Nevertheless, no economic evaluation of strategies to treat lipodystrophy has been undertaken that includes the full range of medical and surgical therapies. Given the cost of leptin, it is suggested that treatment should only be started in liaison with specialised lipodystrophy services, where accessible.

## Improving diagnosis rates of monogenic diabetes

Many people with potentially treatable monogenic beta cell and insulin-resistant diabetes are currently misclassified as having type 2 diabetes. An immediate priority is thus to facilitate identification of patients for genetic testing. A widely used MODY probability calculator that incorporates disease biomarkers and simple clinical data [88, 89] has proven successful in such triage [14]. No such calculator yet exists for monogenic insulin-resistant diabetes, but ‘genetic’ lipodystrophy may occur in up to one in 7000 of the general population [90], suggesting a large unmet need. With the advance of artificial intelligence applied to healthcare data at scale, it is likely that digital approaches to predicting monogenic diabetes will soon become both more refined and ideally applicable to many more monogenic diabetes subtypes.

Any attempt to screen genetically for monogenic diabetes and to intervene based on results will need to take into account the clinical penetrance of gene variants. Fully penetrant, Mendelian, monogenic disease is to some extent a self-fulfilling construct arising from ascertainment of affected patients and families based on their phenotype (in this case diabetes). It has been of great value for early phases of study of genetic disease, but in some cases has become untenable as the spectrum of genetic variation has been revealed by NGS in large populations [91, 92]. In ‘monogenic’ diabetes, too, population studies ascertaining purely by genotype demonstrate weaker associations between genotype and disease than genetic testing with a clinical indication [12, 13]. This suggests that for some types of diabetes we may move away from the notion of mutations that confer disease risk deterministically (i.e. having the gene change always means having diabetes), to the concept that gene changes, even when implicated in monogenic disease, confer disease risk probabilistically (i.e. having the gene mutation increases diabetes probability/expressivity to different degrees) [93].

## Significance of monogenic diabetes for ‘common’ type 2 diabetes

The importance of monogenic disease extends beyond clinically recognisable subtypes nested within diabetes populations, as NGS suggests overlap between monogenic and ‘common’ type 2 diabetes [92]. More than 5% of people with characteristics of type 2 diabetes have been shown to harbour mutations that cause monogenic diabetes (according to standards for interpretation of variants in rare genetic disorders [31]), and in particular MODY [10, 12, 94, 95]). Such people were leaner and developed diabetes earlier than non-carriers with type 2 diabetes [10], but would often not have been detected without genetic screening. None developed type 2 diabetes before 25 years of age, and the first-degree family history of diabetes was similar between carriers and non-carriers [10]. The finding of lipodystrophy causal variants in up to one in 7000 people [90] suggests that a similar paradigm may apply to insulin-resistant diabetes. These discoveries open a gateway for precision medicine among some newly diagnosed patients with apparent type 2 diabetes. Routine screening for monogenic diabetes genes warrants assessment; however, no modelling has been reported, and feasibility of screening followed by appropriate treatment changes requires testing in large-scale studies.

Taking one step further, the possibility that a significant portion of type 2 diabetes heritability may be attributable to rare, functional gene variants has long been mooted. This was encouraged by the limited early success of GWAS focused on commoner gene variants. Sequencing of large populations has indeed identified some rare variants (e.g. in *MTNR1B*, *PPARG*, *SLC30A8*, *HNF1A*, *PDX1*, *PAM*) that confer risk of type 2 diabetes that is intermediate between common SNPs and mutations causing monogenic diabetes [96]. Moreover, in isolated populations, even higher prevalence rates of loss-of-function mutations in *AKT2* and *TBC1D4*, both involved in insulin signalling and implicated in monogenic insulin resistance, have been described [97, 98]. For *TBC1D4* mutation carriers, some evidence for precision exercise intervention has recently been advanced [99].

Nevertheless, as GWAS have grown ever larger, several hundred common polymorphisms associated with type 2 diabetes risk [100] have been identified, mostly located in non-coding DNA regions, and NGS has not supported a major population-wide effect of rare alleles in monogenic diabetes genes [92]. Evidence thus now favours the notion that most type 2 diabetes risk is conferred by aggregated effect of large numbers of common variants of small effect size.

As genetic risk associations of ever smaller effect size proliferate, study of monogenic diabetes may become more important rather than less relevant, however. Understanding of monogenic diabetes syndromes and underpinning mechanisms can inform clustering of common polygenic risk alleles into functionally coherent subgroups. This is valuable given very small effect sizes of common variants, which are difficult to resolve experimentally. For example, learning from monogenic lipodystrophy has informed clustering of traits into lipodystrophic and non-lipodystrophic patterns [101–103], and has been used to suggest functionally distinct groups of common variants.

## Conclusion

Monogenic diabetes is underdiagnosed, yet offers increasing opportunities for genotype-targeted behavioural and pharmacological therapy. Lessons from monogenic disorders are also likely applicable to a subset of people with type 2 diabetes with formes frustes of these conditions, which are difficult to discriminate clinically from type 2 diabetes. Whether systematic genetic screening is warranted remains to be determined. Monogenic diabetes may also be conceptually influential in understanding population propensity to obesity-related diabetes. Full realisation of the potential of genetic medicine in the diabetes clinic will require integration of expertise in rare disease genetics, population genetics, data science and clinical care, and breaking down of some traditional clinical silos. Thus, although genetically informed precision diabetes therapy is not yet quite ready for implementation in "common" type 2 diabetes, it now stands on the threshold. The fruits of more than 30 years of genetic research may soon be harvested for wider benefits in routine practice.

## Supplementary Information

ESM 1 (PPTX 568 kb)

## Abbreviations

FPLD3: Familial partial lipodystrophy type 3
GLP-1: Glucagon-like peptide-1
GWAS: Genome-wide association study
NGS: Next generation sequencing
PI3K: Phosphoinositide 3-kinase
rhIGF-1: Recombinant human IGF-1
TZDs: Thiazolidinediones
