# Supplementary material for: Achievements, prospects and challenges in precision care for monogenic insulin-deficient and insulin-resistant diabetes
Source: Diabetologia. 2022 May 27;65(11):1782–95. doi: 10.1007/s00125-022-05720-7 (PMC9522735; doi:10.1007/s00125-022-05720-7)
Supplement: Supplementary file 1 — (PPTX 568 kb) [file 125_2022_5720_MOESM1_ESM.pptx]

## Slide 1
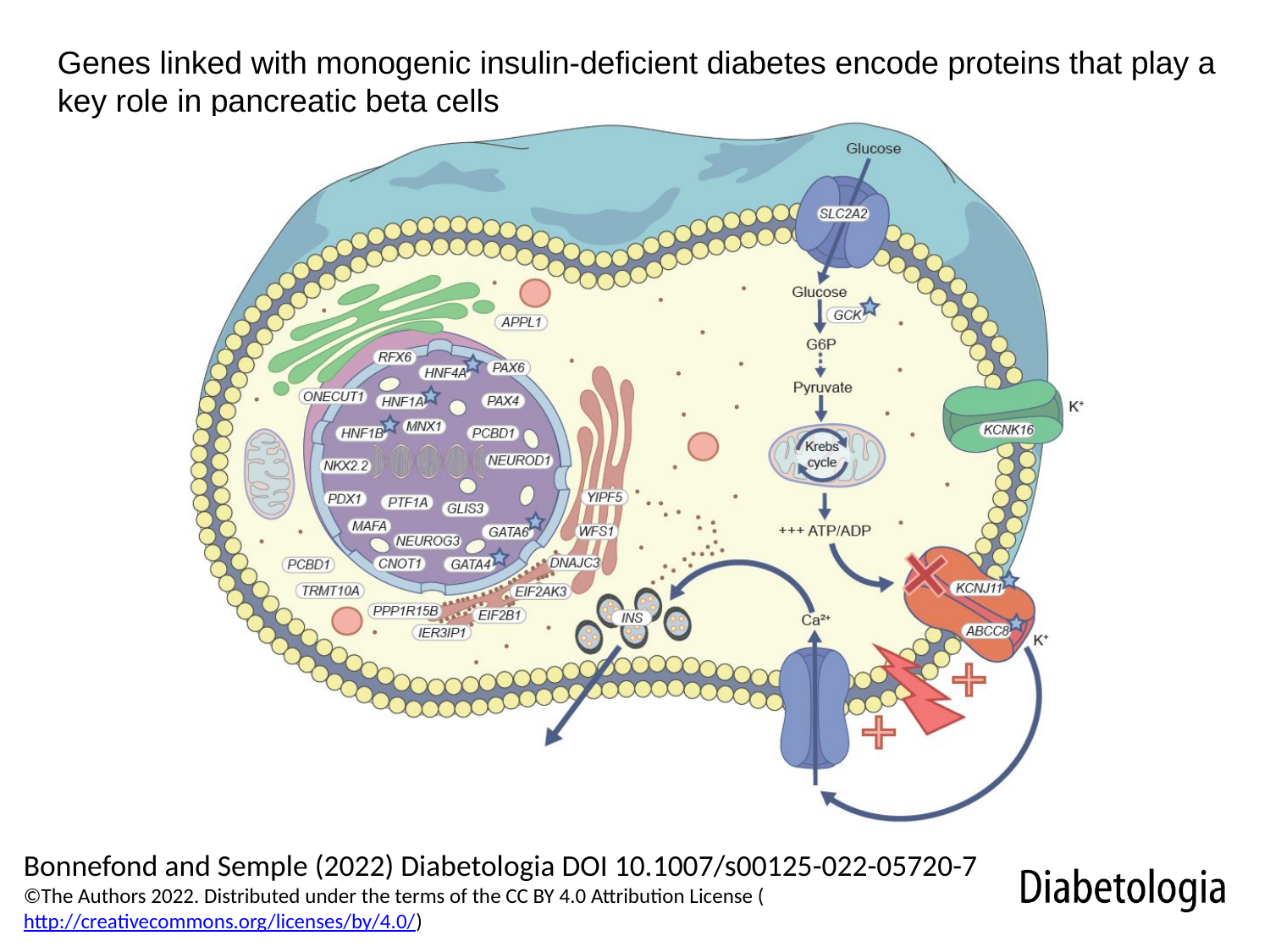

Genes linked with monogenic insulin-deficient diabetes encode proteins that play a key role in pancreatic beta cells
Bonnefond and Semple (2022) Diabetologia DOI 10.1007/s00125-022-05720-7
©The Authors 2022. Distributed under the terms of the CC BY 4.0 Attribution License (http://creativecommons.org/licenses/by/4.0/)

## Slide 2
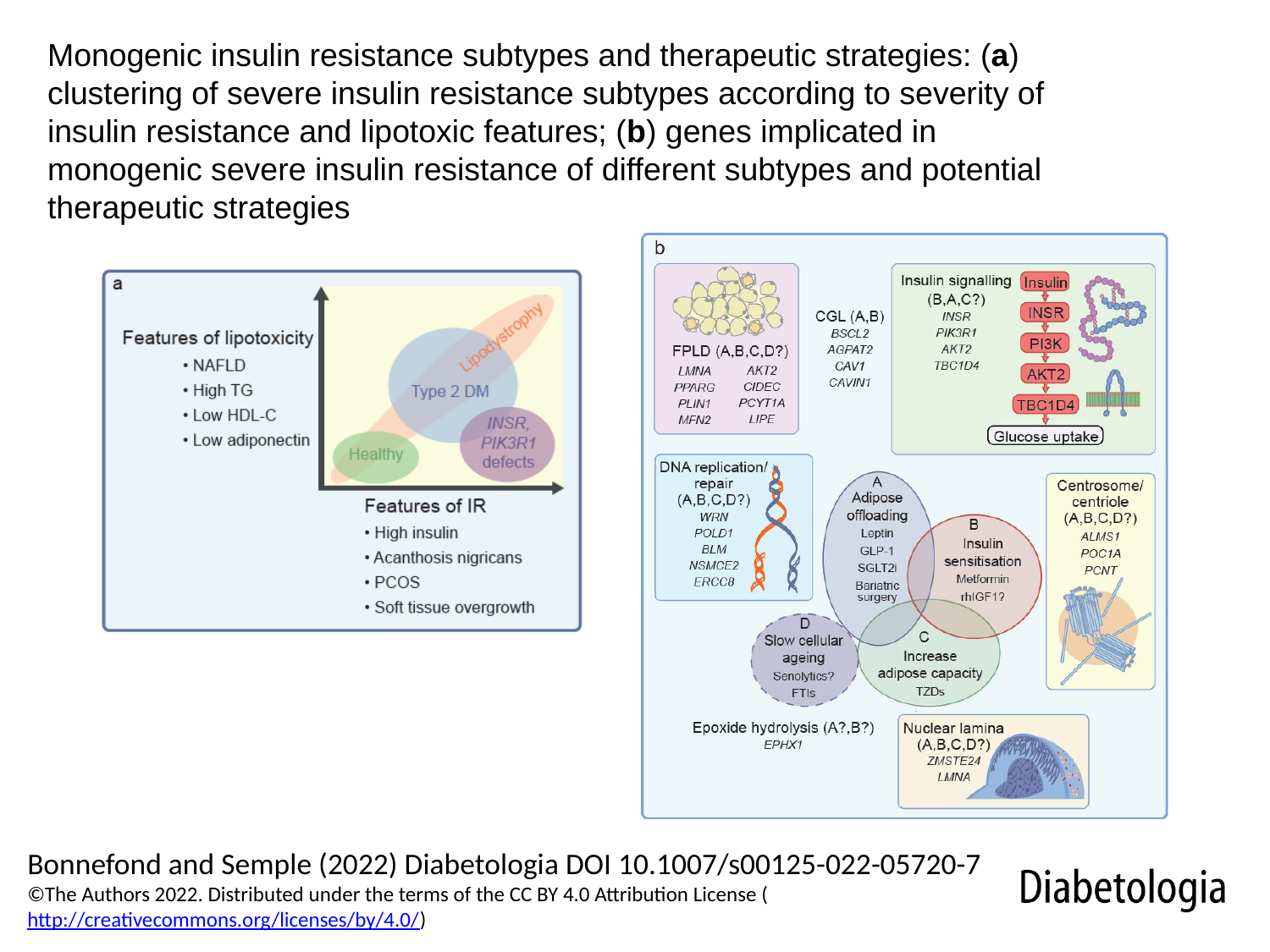

Monogenic insulin resistance subtypes and therapeutic strategies: (a) clustering of severe insulin resistance subtypes according to severity of insulin resistance and lipotoxic features; (b) genes implicated in monogenic severe insulin resistance of different subtypes and potential therapeutic strategies
Bonnefond and Semple (2022) Diabetologia DOI 10.1007/s00125-022-05720-7
©The Authors 2022. Distributed under the terms of the CC BY 4.0 Attribution License (http://creativecommons.org/licenses/by/4.0/)
